# Novel Red-Emitting Eu^3+^-Doped Y_2_(W_x_Mo_1−x_O_4_)_3_ Phosphor with High Conversion Efficiency for Lighting and Display Applications

**DOI:** 10.3390/molecules28124624

**Published:** 2023-06-07

**Authors:** Fan Chen, Muhammad Nadeem Akram, Xuyuan Chen

**Affiliations:** Department of Microsystems, Faculty of Technology, Natural Sciences and Maritime Sciences, Campus Vestfold, University of South-Eastern Norway, 3184 Borre, Norway; chenfan13571038693@163.com (F.C.); muhammad.n.akram@usn.no (M.N.A.)

**Keywords:** Eu^3+^ doped, red phosphor, quantum efficiency, thermal quenching, laser lighting

## Abstract

In this study, a series of trivalent europium-doped tungstate and molybdate samples were synthesized using an improved sol-gel and high-temperature solid-state reaction method. The samples had different W/Mo ratios and were calcined at various temperatures ranging from 800 to 1000 °C. The effects of these variables on the crystal structure and photoluminescence characteristics of the samples were investigated. It was found that a doping concentration of 50% for europium yielded the best quantum efficiency based on previous research. The crystal structures were found to be dependent on the W/Mo ratio and calcination temperature. Samples with x ≤ 0.5 had a monoclinic lattice structure that did not change with calcination temperature. Samples with x > 0.75 had a tetragonal structure that remained unchanged with calcination temperature. However, samples with x = 0.75 had their crystal structure solely dependent on the calcination temperature. At 800–900 °C, the crystal structure was tetragonal, while at 1000 °C, it was monoclinic. Photoluminescence behavior was found to correlate with crystal structure and grain size. The tetragonal structure had significantly higher internal quantum efficiency than the monoclinic structure, and smaller grain size had higher internal quantum efficiency than larger grain size. External quantum efficiency initially increased with increasing grain size and then decreased. The highest external quantum efficiency was observed at a calcination temperature of 900 °C. These findings provide insight into the factors affecting the crystal structure and photoluminescence behavior in trivalent europium-doped tungstate and molybdate systems.

## 1. Introduction

Solid-state lighting (SSL) technology has emerged as a major contender in the field of artificial lighting, replacing traditional incandescent and fluorescent lamps due to its advantages in terms of high efficiency, small size, longer life span, cost-effectiveness, and eco-friendliness [1,2,3,4,5,6]. The key component of this technology is the white light-emitting diode (WLED), which is fabricated using inorganic semiconductor electro-optical conversion chips [7,8,9]. Two primary approaches are used for generating white light: one based entirely on semiconductor chips, and another utilizing semiconductor chips along with wavelength conversion materials (phosphors) [10,11,12]. In the former approach, LED chips of different colors (wavelengths) are mixed to produce white light. The latter method involves coating blue or near-ultraviolet (NUV) LEDs with a phosphor that absorbs and converts blue/NUV light into visible light of a different wavelength, which is then combined to generate white light [13]. The first commercially available LED plus wavelength-conversion material method utilized a blue-emitting GaN-based LED chip combined with a yellow-emitting phosphor (YAG: Ce^3+^) to create white light [14]. However, this approach has certain limitations, primarily a low color rendering index (CRI), particularly in R9 (usually negative) that represents red, owing to the absence of red components in the resulting white light spectrum [15,16]. To enhance CRI, a mixture of different phosphor materials is typically employed; specifically, red-emitting phosphors are added to yellow-emitting phosphors [17,18]. Thus, extensive research efforts have been directed towards the development of high-quality and cost-effective red phosphor materials [19,20].

Rare-earth ions are commonly used as the luminescence center of red-emission materials, with Sm^3+^, Eu^3+^, Tb^3+^, and Dy^3+^ being the most prevalent. Eu^3+^ is particularly known for its intense emission, high efficiency, and saturated red color [6,21,22]. The electronic configuration of Eu^3+^ can be expressed as [Xe]4f^6^, with 54 electrons in the same closed shell as the xenon atom and 6 electrons in the 4f shell [23]. The 4f shell is located within the closed 5s^2^ and 5p^6^ shells and is adequately shielded from the crystal field environment. This leads to a largely unaffected energy level distribution of the 4f shell by an external environment. Molybdates and tungstates are classic host materials for Eu^3+^-doped red-emission phosphor materials, extensively studied by scientific researchers. Due to having similar chemical properties and physical structures, Mo and W can be doped into each other’s group ([MoO_4_]^2−^ and [WO_4_]^2−^) without any changes to the crystal structure [24,25,26,27]. However, the crystal lattice parameters and the internal stress of the mixed structure, (Mo/WO_4_)^2−^, could vary slightly due to the difference in size of Mo^6+^ and W^6+^ ions. Depending on the synthesis conditions, such as calcination temperature and pressure, different types or mix of crystalline structures, such as monoclinic (C2/c), orthorhombic (Pba2, Pbcn), and tetragonal (P$\bar{4}$2_1_m), could possibly be created in the material.

Yttrium molybdate, as the main material of trivalent europium, has been reported multiple times [28,29,30]; in order to further improve the photoluminescence performance of this type of powder material, we replaced some molybdenum atoms with tungsten atoms to form a new host material, Y_2_(W_x_Mo_1−x_O_4_)_3_. In this study, a series of Eu^3+^-doped Y_2_(W_x_Mo_1−x_O_4_)_3_ phosphors were successfully synthesized using improved sol-gel and high-temperature solid-state reaction methods. The impact of synthesis temperature and Mo/W ratio on the lattice structure was assessed, together with the photoluminescence performance of samples with different lattice structures. The novel red phosphor Eu^3+^: Y_2_(W_x_Mo_1−x_O_4_)_3_ presented excellent photoluminescence performance, particularly in terms of luminescence intensity and thermal quenching temperature, making this material suitable for improving the color rendering index of white LEDs.

## 2. Results and Discussion

### 2.1. Morphology and Particle Size by Scanning Electron Microscopy

**Figure 1:**
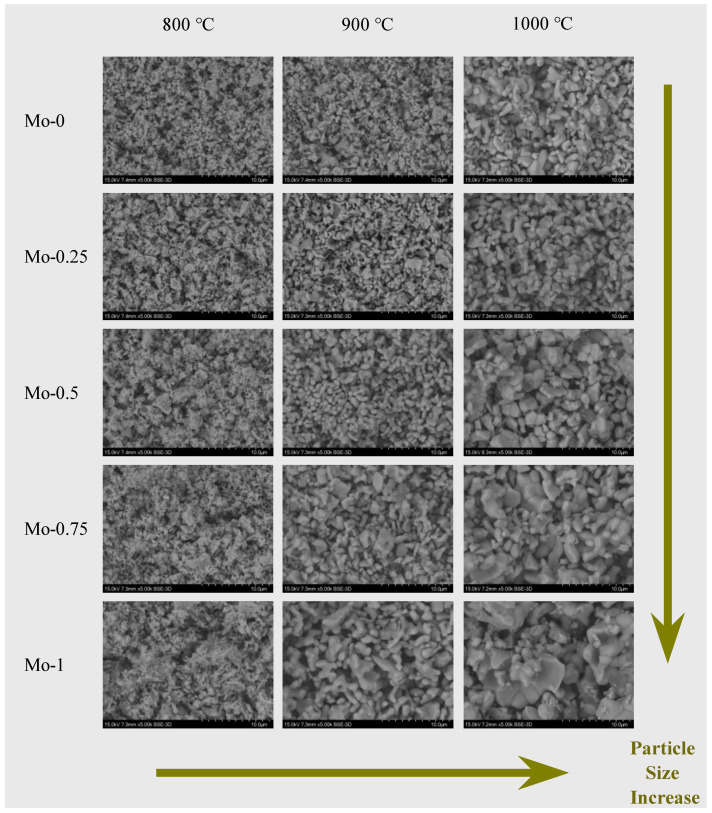
SEM images of samples at different calcination temperatures and different Mo/W ratios.

The present study investigates the particle size and microscopic morphology of synthesized Eu^3+^-doped tungstate/molybdate red phosphor powder. Scanning electron microscopy (SEM) was utilized to obtain the SEM images of Y_2_(W_x_Mo_1−x_O_4_)_3_ (x = 0, 0.25, 0.50, 0.75, and 1.0) samples calcined at different temperatures (800 °C, 900 °C, and 1000 °C). The SEM images revealed that the powder particles have a homogenous particle size, irregular near-round or oval-shaped grains, and a clear grain boundary with some agglomerations, as shown in Figure 1. The particle size is observed to be influenced by the combined effect of calcination temperature and x value. At a calcination temperature of 800 °C, the particle size remains constant at around 0.5 µm regardless of the variation in x value. Conversely, when the calcination temperature is greater than 800 °C, the particle size significantly increases with the increase in Mo content, ranging from 0.7 to 3 µm (900 °C) and from 1 to 5 µm (1000 °C).

### 2.2. Component Identification by Energy Dispersive Spectrometer

**Figure 2:**
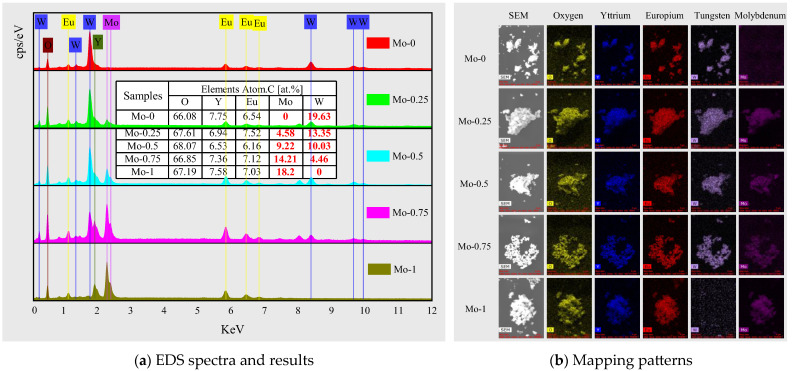
EDS energy spectra (**a**) and mapping patterns (**b**) of samples with different Mo content at 900 °C calcination temperature; the table in figure (**a**) shows element content of each sample.

The elemental composition and proportion of each component were analyzed and confirmed through energy-dispersive spectroscopy (EDS), as presented in Figure 2a. The interpolation table displays the percentage of atomic relative quantity of each element in each sample. In this research, Eu^3+^ was used as the luminescent center and was doped into Y_2_(W_x_Mo_1−x_O_4_)_3_ (x = 0, 0.25, 0.50, 0.75, and 1.0) host material to replace half of the yttrium atom position, such that the Eu^3+^ doping concentration was expected to be 5.9 mol%. Based on the EDS results, the molar concentration of Eu^3+^ doping is 6–7%, slightly higher than the design value of 5.9%. However, this error falls within the typical error range of EDS for element content measurement; thus, the EDS measurement results are reliable. The manual shielding of the detected carbon peak during quantitative analysis of EDS leads to a slightly higher content of other elements. The x value was calculated based on the molybdenum and tungsten content measured by EDS, and these values (0, 0.255, 0.479, 0.761, and 1) were found to be close to the design value, confirming the successful synthesis of a series of Eu^3+^-doped Y_2_(W_x_Mo_1−x_O_4_)_3_ (x = 0, 0.25, 0.50, 0.75, and 1.0) red-emitting materials. The EDS mapping patterns in Figure 2b support this finding.

### 2.3. Crystal Structures and XRD Patterns

Trivalent yttrium/europium molybdates and tungstates belong to the RE_2_(MO_4_)_3_ family and can adopt three distinct crystal structures, which are influenced by composition and synthesis temperature [31,32,33,34]. These include the monoclinic C12/c1 space group of the high-temperature β phase, the tetragonal P$\bar{4}$2_1_m space group of the high-temperature α phase, and the orthorhombic Pba2/Pbcn space group of the metastable β’ phase [28,29]. Moisture content during the synthesis process also affects the crystal structure, particularly in the Pba2/Pbcn phase. Studies show that Pbcn with six coordination is kinetically favored over Pba2 with seven coordination, but exposure to humid environments or annealing below 550 °C can transform Pbcn into Pba2 due to its thermodynamic and hygroscopic stability [30,35].

**Figure 3:**
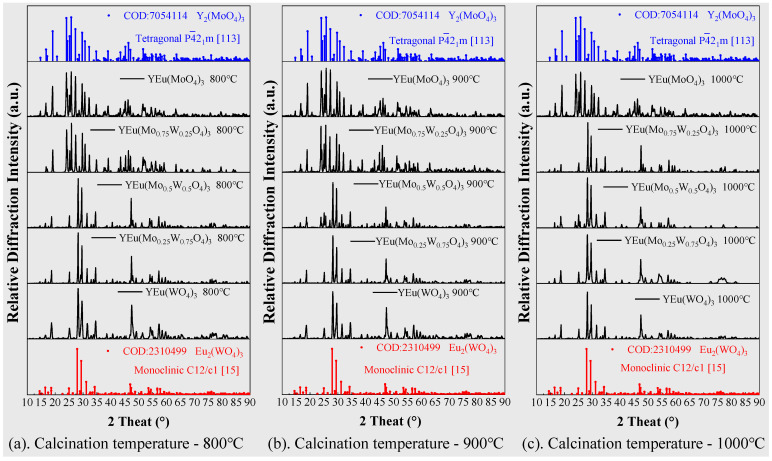
XRD diffraction patterns of the samples at different calcination temperatures with different ratios of Mo/W ((**a**) 800 °C, (**b**) 900 °C and (**c**) 1000 °C).

**Figure 4:**
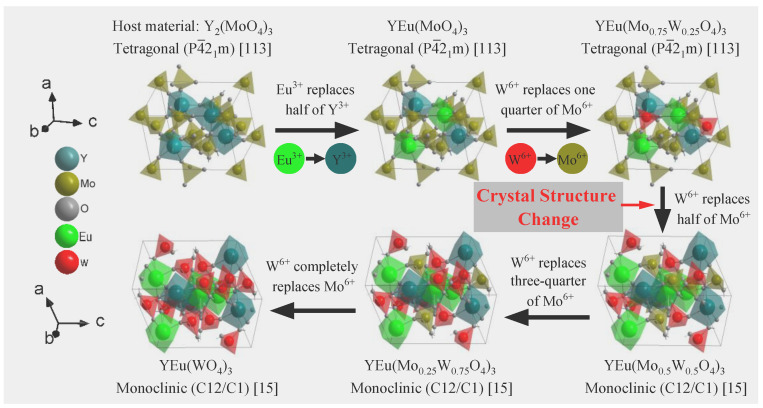
The crystal structure of the sample changes with the variable composition at 800 and 900 °C calcination temperature.

To investigate the luminescent properties of Y_2_(MoO_4_)_3_, we synthesized it using the method described in Section 3.1 to ultimately form the tetragonal P$\bar{4}$2_1_m space group. Trivalent europium serves as the red luminescent center and replaces half of the trivalent yttrium ions. Our XRD results showed that doping Eu^3+^ did not alter the crystal structure, nor did calcination at different temperatures. To improve luminescence, we replaced some molybdenum with tungsten to form [W_x_Mo_1−x_O_4_]^2−^ (x = 0, 0.25, 0.50, 0.75, and 1.0). Our XRD diffraction pattern in Figure 3, shows that increasing the proportion of W^6+^ atoms replacing Mo^6+^ can lead to a change in crystal structure from tetragonal to monoclinic. This concentration was influenced by calcination temperature, where the W^6+^ concentration causing crystal change was 0.5 below 1000 °C and 0.25 at 1000 °C. Figure 4 shows the crystal structure’s change with the increase in W^6+^ content, while calcination temperature remains below 1000 °C. 

### 2.4. Excitation and Emission Spectra

**Figure 5:**
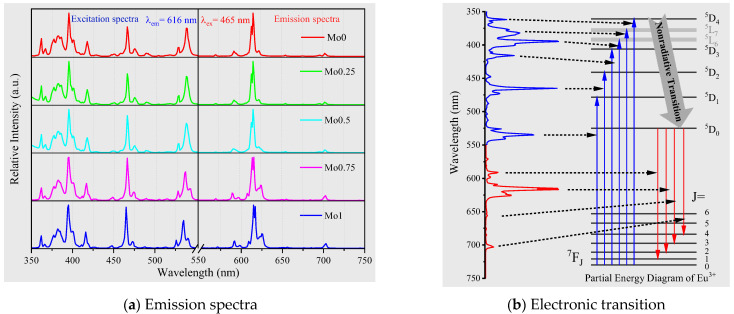
Excitation and emission spectra comparison of samples at the same calcination temperature (900 °C) and different Mo/W ratios (**a**). Correspondence between emission spectrum and electronic transition (**b**).

The present study investigates the excitation and emission spectra of Eu^3+^-doped Y_2_(W_x_Mo_1−x_O_4_)_3_ (x = 0, 0.25, 0.50, 0.75, and 1.0) at room temperature. Excitation spectra were examined at an emission wavelength of 616 nm, while emission spectra were observed under an excitation wavelength of 465 nm. The results reveal that the calcination temperature does not influence the relative excitation and emission spectra. Therefore, analysis was restricted to the sample with a calcination temperature of 900 °C. It is well established that the excitation spectrum of Eu^3+^-doped red emission material comprises an intense broad band at 230–350 nm due to the charge transfer from ligand (O^2−^) to metal (Eu^3+^ and Mo^6+^) and many sharp lines at 350–500 nm, ascribed to typical Eu^3+^ electron transition 4f-4f. However, the ultraviolet broadband excitation peak is seldom applicable in practical applications because of the large release of heat during the Stokes shift. Therefore, the present study focuses on the narrow-band excitation peaks of Eu^3+^ and carefully distinguishes the energy level transitions of each excitation peak, as depicted in Figure 5.

The present study highlights the photoluminescence spectra of the characteristic f-f transition in the 4f^6^ configuration of Eu^3+^ ion in the compound, as illustrated in Figure 5a, divided into excitation spectrum and emission spectrum. The narrow excitation peak, ranging from 350 nm to 550 nm, originates from the electronic transitions of Eu^3+^ from ^7^F_0_ ground state to various excited states, viz., ^5^D_4_ (360 nm), ^5^L_7_ (380 nm), ^5^L_6_ (395 nm), ^5^D_3_ (415 nm), ^5^D_3_ (465 nm), and ^5^D_1_ (535 nm) [36,37,38]. Among them, ^7^F_0_→^5^L_6_ (395 nm) and ^7^F_0_→^5^D_3_ (465 nm) exhibit strong excitation intensity, which synchronizes adequately with the output wavelength of near-UV and blue LED/LD chips produced on a large scale [39,40]. The emission spectra are situated in the 550–750 nm range, found to be linear in spectrum and correspond to the electronic transitions of Eu^3+^ from ^5^D_0_ to ^7^F_1_, ^7^F_2_, ^7^F_3_, and ^7^F_4_ [41,42,43]. The ratio of the integrated area of the emission peaks corresponding to the ^5^D_0_→^7^F_2_ and ^5^D_0_→^7^F_1_ transitions is calculated to be around 7.8, so it can be concluded that Eu^3+^ is at a very low symmetry site in the system [44,45]. The most prominent emission peak is ^5^D_0_→^7^F_2_ (616 nm), which appears as a dazzling red color.

### 2.5. Quantum Efficiency 

In the context of evaluating the photoluminescence intensity and heating situation in phosphor materials, quantum efficiency stands as a crucial criterion. We conducted a measurement of the quantum efficiency of the Eu^3+^-doped Y_2_(W_x_Mo_1−x_O_4_)_3_ (x = 0, 0.25, 0.50, 0.75, and 1.0) samples at room temperature. The measurement was carried out utilizing a 465 nm laser diode, a calibrated spectrometer, and a standard integrating sphere. As part of the process, we measured and calculated the number of excited light photons emanating from the excitation light source, the number of photons absorbed by the sample, and the number of photons emitted by the sample. Quantum efficiency was computed via the utilization of the following formulae, i.e., Formula (1) and (2).

(1) $$\eta_{IQE}=\frac{\int R_{Emit}}{\int B_{Abs}}$$

(2) $$\eta_{EQE}=\frac{\int R_{Emit}}{\int B_{Provid}}$$

The calculation requires the determination of $\int R_{Emit}$, which stands for the number of photons emitted by the sample; $\int B_{Abs}$, which stands for the number of photons absorbed by the sample; and $\int B_{Provid}$, which represents the number of excited light photons provided by the excitation light source. Among the calculated values, $\eta_{IQE}$ has been introduced as the internal quantum efficiency, which is a reliable indicator of the sample’s ability to convert excitation light energy into emission light. Notably, a higher value of $\eta_{IQE}$ indicates a higher efficiency of the sample in emitting light [46,47]. Conversely, a higher value of $\eta_{IQE}$ implies a higher thermal efficiency. Additionally, $\eta_{EQE}$ denotes the external quantum efficiency, which reflects the actual photoluminescence strength of phosphor materials. It combines the internal quantum efficiency and absorption efficiency measures.

The study investigates the influence of calcination temperature on the quantum efficiency of Eu^3+^-doped Y_2_(W_x_Mo_1−x_O_4_)_3_ powders, where x varies from 0 to 1 in increments of 0.25. The results are presented in Table 1. The quantum efficiency measured results of Eu^3+^-doped Y_2_(W_x_Mo_1−x_O_4_)_3_ (x = 0, 0.25, 0.50, 0.75, and 1.0) reveal a decreasing trend in $\eta_{IQE}$, while the $\eta_{EQE}$ initially increases and then decreases with an increase in calcination temperature. The observed reduction in $\eta_{IQE}$ is attributed to two factors: (i) a decrease in the escape rate of excitation luminescence due to an increase in grain size, and (ii) an increase in surface defects. On the other hand, an increase in powder volume and the number of luminescent centers contributes to the enhancement of $\eta_{IQE}$. Interestingly, the tetragonal crystal structure exhibits higher quantum efficiency than the monoclinic crystal structure [48]. Based on a comprehensive analysis of the quantum efficiency of these samples, the Eu^3+^-doped Y_2_(Mo_0.75_W_0.25_O_4_)_3_ powder calcined at 900 °C exhibits the highest quantum efficiency, with an $\eta_{EQE}$ value of 0.32. This is much higher than the reported $\eta_{EQE}$ of similar materials, such as 0.2238 (Y_2_(MoO_4_)_3_:Eu^3+^) [30], 0.1278 (CaMoO_4_:Eu^3+^) [49], and 0.1 (Y_2_(MoO_4_)_3_:Eu^3+^/Au) [29]. These findings provide valuable insights into optimizing the synthesis conditions of luminescent materials for potential applications in optoelectronic devices. 

### 2.6. Thermal Quenching

**Figure 6:**
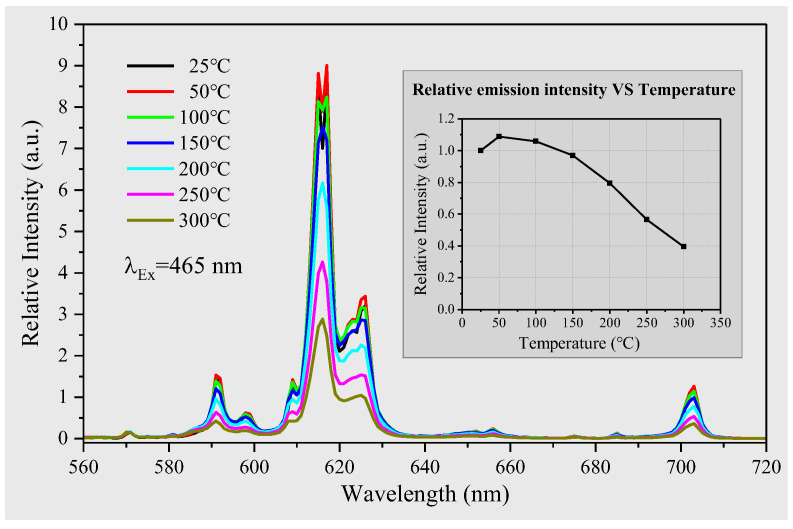
The emission spectra thermal quenching behavior of YEu(Mo_0.75_W_0.25_O_4_)_3_ recorded under 465 nm; the illustration shows that the integral value of emission spectrum changes with the increase in temperature.

The phenomenon of temperature-dependent changes in the intensity of phosphor luminescence is a common occurrence in many industries [50,51]. It is widely accepted that the quenching limit for phosphor materials is reached when the luminescence intensity decreases to 80% of its value at room temperature with an increase in temperature. This temperature is known as the quenching temperature of the phosphor material. In this study, we present the measurement results for the quenching temperature of a sample of Eu^3+^-doped Y_2_(Mo_0.75_W_0.25_O_4_)_3_, as shown in Figure 6. Since the thermal quenching behavior of all samples is similar, we focused our analysis on the representative case of Mo_0.75_. Our findings provide valuable insights into the quenching temperature behavior of this particular phosphor material, which has important implications for future research in the field.

In this study, we conducted emission spectra measurements on a sample under 465 nm excitation light, while increasing the working temperature in steps of 50 °C from 25 °C to 300 °C. Our findings indicate that there is no discernible effect of temperature on the energy levels of Eu^3+^ ions, as evidenced by the lack of any change in the emission peaks position with increasing temperature. To explore the relationship between the integrated emission spectrum values of the Mo_0.75_ sample and working temperature, we generated the illustrated results shown in Figure 6. Our analysis reveals that the luminescence intensity of Mo_0.75_ sample first increases and then decreases with the increase in temperature. At around 50 °C, the luminescence intensity reaches its maximum, corresponding to a value of 1.1 times that at room temperature. This reason is that the probability of the electrons transition from the ground state to the excited state increases with the increase in temperature (the intensification of the thermal movement). However, as the temperature continues to rise, the luminescence intensity subsequently decreases. By the time the temperature reaches 150 °C, the luminescence intensity has essentially returned back to its original level at room temperature. The thermal quenching temperature of the sample was found to be 200 °C since at this point, the luminescence intensity had decreased to 80% of its value at room temperature. These results provide valuable insights into the precise mechanisms underlying the temperature-dependent behavior of phosphor material, which will help guide future research and applications in this field.

The phenomenon of thermal quenching is attributed to the absorption of heat energy by the electron in its excited state. This process leads to a transition to a higher energy level, the CTB (charge transfer band), which offers a nonradiative pathway for the electron [52,53]. Upon reaching the ground state, more heat is released, and this further increases the temperature, thereby creating a vicious cycle reminiscent of an avalanche effect. Elevating the working temperature of the phosphor material enhances the conversion of excitation energy into thermal energy, exacerbating the avalanche effect, and eventually leading to the triggering of the quenching temperature [50]. At this point, the temperature of the phosphor material rises rapidly, and its emission is quenched. These insights into the physical mechanism of thermal quenching have significant implications for understanding the behavior of phosphor materials under changing temperature conditions and can guide future developments in this field [54].

## 3. Materials and Methods

### 3.1. Synthesis

This study reports on the synthesis of a series of red phosphor powder samples, which are doped with Eu^3+^ in Y_2_(W_x_Mo_1−x_O_4_)_3_ (x = 0, 0.25, 0.50, 0.75, and 1.0), through a combination of an improved sol-gel process and high-temperature solid-state reaction method. The proportion of Eu^3+^ replacing Y^3+^ is 50%, and the molar concentration is about 6%. In the sol-gel process, citric acid was chosen as the chelating agent, with its molar amount being equivalent to the molar number of cations in the solution. The suspension solution was formed by adding yttrium nitrate (III), ammonium molybdate, europium nitrate (III), and tungstic acid into the citric acid solution. After being heated and stirred for 12–15 h, the water in the solution was evaporated, leaving a semi-solid gel behind. Subsequently, the semi-solid gel underwent calcination in a muffle furnace, experiencing solid-state reactions at high temperatures to form the desired red phosphor materials. These synthesized phosphors have potential applications in the field of solid-state lighting technology driven by their excellent photoluminescence properties.

### 3.2. Characterization

We investigated the morphology, particle size, composition, and crystal structures of our samples through the use of Hitachi SU3500 for obtaining SEM images and EDS spectra. Our identification of crystal structures was based on X-ray diffraction patterns obtained from an Equinox1000 Sn.1612EQ1000137 diffractometer (Thermo Fisher; Horten, Norway), which utilized Cu Kα radiation (λ = 1.5418 Å). Furthermore, we measured the emission and excitation spectra using an Edinburgh FS05 Fluorescence Spectrometer, and determined the quantum efficiency via a calibrated AvaSpec-ULS2048-EVO PL spectrometer and AvaSphere-50 integrating sphere. To assess thermal quenching temperature, our samples were heated with a HT24S-24W metal ceramic heater (ThorLabs). Photoluminescence relative intensity readings were taken at varying temperatures using the Edinburgh FS05 fluorescence spectrometer.

## 4. Conclusions

The synthesis of novel red phosphor powder materials, Eu^3+^:Y_2_(W_x_Mo_1−x_O_4_)_3_ (x = 0, 0.25, 0.50, 0.75, and 1.0), was successfully achieved using an improved sol–gel process in combination with a high-temperature solid-state reaction method. The lattice structure of the samples was studied as a function of synthesis temperature and Mo/W ratio, and the photoluminescence performance of the resulting powders with varying lattice structures was evaluated. The monoclinic lattice structure was found to persist for x ≤ 0.5 regardless of calcination temperature, while the tetragonal crystal structure remained unchanged for x > 0.75 at all calcination temperatures studied. For x = 0.75, the crystal structure transformed from tetragonal (800–900 °C) to monoclinic (1000 °C) depending on the calcination temperature. The photoluminescence efficiency of the tetragonal crystal structure was ~5% higher than that of the monoclinic crystal structure. A comprehensive analysis of the quantum efficiency of the samples showed that the Eu^3+^-doped Y_2_(Mo_0.75_W_0.25_O_4_)_3_ sample calcined at 900 °C exhibited the highest quantum efficiency, with an external quantum efficiency of up to 0.32. In addition, the sample’s measured thermal quenching temperature was found to be 200 °C, indicating that this material is highly promising for use in harsh environments and can withstand high excitation power density. These findings suggest that the new material could serve as a crucial component in the next generation of lighting technology, including laser lighting.

## Figures and Tables

**Table 1:** The quantum efficiency measured results of Eu^3+^-doped Y_2_(W_x_Mo_1−x_O_4_)_3_ (x = 0, 0.25, 0.50, 0.75, and 1.0).

Samples	800 °C		900 °C		1000 °C	
	$\eta_{IQE}$	$\eta_{EQE}$	$\eta_{IQE}$	$\eta_{EQE}$	$\eta_{IQE}$	$\eta_{EQE}$
Mo1	0.95	0.21	0.93	0.25	0.91	0.26
Mo0.75	0.95	0.2	0.92	0.32	0.84	0.28
Mo0.5	0.91	0.13	0.88	0.16	0.85	0.14
Mo0.25	0.9	0.12	0.89	0.17	0.84	0.13
Mo0	0.9	0.11	0.88	0.15	0.85	0.14

## Data Availability

More research data are available from the authors on request.

## Sample Availability

The reported samples are available from the authors on request.
